# The role of membrane ERα signaling in bone and other major estrogen responsive tissues

**DOI:** 10.1038/srep29473

**Published:** 2016-07-08

**Authors:** K. L. Gustafsson, H. Farman, P. Henning, V. Lionikaite, S. Movérare-Skrtic, J. Wu, H. Ryberg, A. Koskela, J.-Å. Gustafsson, J. Tuukkanen, E. R. Levin, C. Ohlsson, M. K. Lagerquist

**Affiliations:** 1 Centre for Bone and Arthritis Research, Department of Internal Medicine and Clinical Nutrition at Institute of Medicine, Sahlgrenska Academy, University of Gothenburg, SE-41345 Gothenburg, Sweden; 2 Unit of Cancer Research and Translational Medicine, MRC Oulu and Department of Anatomy and Cell Biology, University of Oulu, FI-90014 Oulu, Finland; 3 Center for Nuclear Receptors and Cell Signaling, Department of Biology and Biochemistry, University of Houston, Houston, Texas, 77204-5056, USA; 4 Division of Endocrinology, Veterans Affairs Medical Center, Long Beach, California, CA90822, USA; 5 Department of Developmental and Cell Biology, Department of Medicine, and Department of Biochemistry, University of California, Irvine, California, CA92717, USA

## Abstract

Estrogen receptor α (ERα) signaling leads to cellular responses in several tissues and in addition to nuclear ERα-mediated effects, membrane ERα (mERα) signaling may be of importance. To elucidate the significance, *in vivo*, of mERα signaling in multiple estrogen-responsive tissues, we have used female mice lacking the ability to localize ERα to the membrane due to a point mutation in the palmitoylation site (C451A), so called Nuclear-Only-ER (NOER) mice. Interestingly, the role of mERα signaling for the estrogen response was highly tissue-dependent, with trabecular bone in the axial skeleton being strongly dependent (>80% reduction in estrogen response in NOER mice), cortical and trabecular bone in long bones, as well as uterus and thymus being partly dependent (40–70% reduction in estrogen response in NOER mice) and effects on liver weight and total body fat mass being essentially independent of mERα (<35% reduction in estrogen response in NOER mice). In conclusion, mERα signaling is important for the estrogenic response in female mice in a tissue-dependent manner. Increased knowledge regarding membrane initiated ERα actions may provide means to develop new selective estrogen receptor modulators with improved profiles.

Estrogens are classically considered reproductive hormones, but they also induce cellular responses in several non-reproductive tissues and are important for the overall health of women. The importance of estrogen for skeletal health is well known and an important research area since estrogen deficiency, caused by ovarian failure at menopause, is a major risk for development of osteoporosis and leads to increased fracture risk^1^. Estrogen treatment prevents this increased fracture risk, but is associated with side effects, such as increased risk of cancer in reproductive organs and thromboembolism^2^,^3^. Thus, increased knowledge regarding signaling pathways underlying the effects of estrogen in various tissues would aid in the search for tissue-specific estrogen treatment options.

Estrogens exert effects via binding to estrogen receptors (ERs), where ERα is considered an important ER in many tissues, including bone^4^,^5^,^6^, while ERβ is of great significance in e g the CNS and the hematopoietic system and has been shown to slightly modulate ERα action in the female skeleton^7^,^8^,^9^,^10^,^11^. Estrogenic effects via ERα are mediated by different signaling pathways. The genomic effects involve translocation of the estrogen-ERα complex into the nucleus and either direct binding to estrogen response elements in regulatory sequences of target genes (classical pathway), or binding to other transcription factors (non-classical pathway) and subsequent regulation of gene transcription. In addition to these genomic effects, it is now well established that estrogen exerts non-genomic effects, which are rapid effects that do not involve nuclear localization of the ERs^12^,^13^. The first studies evaluating non-genomic estrogen effects *in vivo* used estrogen dendrimer conjugate (EDC), a macromolecule incapable of entering the nucleus and thereby only able to initiate non-genomic estrogen signaling^14^. These studies demonstrated that non-genomic estrogen signaling can promote cardiovascular protection^15^, mediate neuroprotective effects^16^ and also prevent cortical, but not trabecular, bone loss after estrogen deprivation in female mice^17^.

It has been demonstrated both *in vitro* and *in vivo* that a pool of ERs is situated in the membrane of cells and therefrom initiate non-genomic estrogenic effects^18^,^19^,^20^,^21^. This membrane-bound fraction is estimated to be approximately 5–10%, depending on cell-type^12^. ERα localizes to the membrane via binding to caveolin-1, and a posttranslational modification, i.e. addition of palmitic acid to C451 (C447 in humans), is required for this subcellular localization^22^. To evaluate the importance of membrane localization of ERα *in vivo*, mouse knock-in models, where a cysteine 451-to-alanine mutation is inserted into the ERα locus (esr1), have been generated^20^,^23^. Analyses of these mouse models, named Nuclear-Only Estrogen Receptor (NOER) mice, have shown that loss of membrane initiated ERα signaling leads to female infertility associated with abnormalities in ovarian function and disturbed sex steroid levels^20^,^23^.

In order to evaluate the role of membrane initiated ERα signaling *in vivo* for estrogenic effects in the skeleton and multiple other estrogen responsive tissues, without confounding high sex steroid levels, we have evaluated the estrogen response in adult ovariectomized (ovx) NOER mice.

## Materials and Methods

### Animals

All experimental procedures involving animals were approved by the Ethics Committee at the University of Gothenburg and carried out in accordance with relevant guidelines. Transgenic NOER mice with a point mutation in ERα at the palmitoylation site C451 have been described before^20^. The primers used for genotyping of NOER mice were 5′-CTAAACAAGCTTCAGTGGCTCCTAG-3′ and 5′- ACCTGCAGGGAGAAGAGTTTGTGGC-3′. The mice were housed in a standard animal facility under controlled temperature (22 °C) and photoperiod (12 h of light and 12 h of darkness) and fed phytoestrogen free pellet diet ad libitum (Harlan 2016). Gonadal intact female mice were killed at twelve or sixteen weeks of age. In the treatment experiment, twelve-week-old female mice were ovariectomized (ovx) and treated with a subcutaneous slow-release pellet (60-day-release pellet, Innovative Research of America) with 17β-estradiol (E2) (16,7 ng·mouse^−1^·day^−1^) or placebo for four weeks. Surgery was performed under anesthesia with isoflurane (Baxter Medical AB, Kista, Sweden) and Rimadyl (Orion Pharma AB, Animal Health, Sollentuna, Sweden) was given postoperatively as an analgesic. At termination the mice were anesthetized with Ketanest/Dexdomitor (Pfizer/Orion Pharma), bled, and euthanized by cervical dislocation. Uterus, fat depots, liver and thymus were collected and weighed. The long bones and vertebras were dissected and stored for further analysis.

### Western Blot

Western Blot and protein preparation of uteri and bone from NOER and WT mice were performed as previously described^24^. Briefly, tissues were homogenized in RIPA-buffer supplemented with complete Mini EDTA-free Protease Inhibitor Cocktail (Roche Diagnostics). The rabbit polyclonal ERα antibody (MC-20; Santa Cruz Biotechnology), diluted 1:1000, was used^24^. An anti-rabbit HRP-conjugated secondary antibody (GE Healthcare), diluted 1:10,000, and Clarity Western ECL substrate (BioRad), were used to visualize the bands.

### Real-Time PCR

RNA was isolated from uterus, vertebral bodies L_3_ and L_6_ (trabecular bone) and the mid-diaphyseal cortical bone from long bones (tibia and femur) using TRIzol reagent (Sigma) followed by the RNeasy Mini Kit (Qiagen). Amplifications were performed using the Applied Biosystem StepOnePlus Real-Time PCR System (PE, Applied Biosystems) and Assay-on-Demand primer and probe sets (PE, Applied Biosystems), labeled with the reporter fluorescent dye FAM. Predesigned primers and probe labeled with the reporter fluorescent dye VIC, specific for 18S ribosomal RNA, were included in the reaction as an internal standard. The assay identification numbers were; ERα: Mm00433147_m1, ERβ: Mm00599819_m1.

### Serum Analyses

Serum levels of 17β-estradiol (E2) and testosterone were measured in a single run by GC-MS/MS, as described previously^25^. As a marker of bone resorption, serum levels of C-terminal type I collagen fragments were assessed using an ELISA RatLaps kit (CTX, Immunodiagostic Systems) according to the manufacturer’s instructions. Serum levels of osteocalcin (OCN), a marker of bone formation, were determined with a mouse osteocalcin immunoradiometric assay kit (Immutopics). Serum leptin levels were measured using a Mouse Leptin ELISA kit (Crystal Chem).

### Assessment of Bone Parameters

#### Dual-Energy X-Ray Absorptiometry (DXA)

Analyses of total body areal bone mineral density (aBMD) and lumbar spine (L_2_-L_5_) aBMD were performed using a Lunar PIXImus mouse densitometer (Wipro GE Healthcare).

#### High-Resolution Microcomputed Tomography (μCT)

High-resolution microcomputed tomography (μCT) analysis was performed on the distal femur and vertebrae L_2_ using an 1172 model μCT (Bruker MicroCT, Aartselaar, Belgium) as previously described^26^. The cortical measurements in the femur were performed in the mid-diaphyseal region of femur starting at a distance of 5.2 mm from the growth plate and extending a further longitudinal distance of 134 μm in the proximal direction. The trabecular bone proximal to the distal growth plate was selected for analyses within a conforming volume of interest (cortical bone excluded), commencing at a distance of 650 μm from the growth plate and extending a further longitudinal distance of 134 μm in the proximal direction. Cortical bone in the vertebral body (L_2_) caudal of the pedicles was selected for analyses within a conforming volume of interest commencing at a distance of 4.5 μm caudal of the lower end of the pedicles, and extending a further longitudinal distance of 225 μm in the caudal direction. For bone mineral density analysis, the equipment was calibrated with ceramic standard samples.

#### Bone Histomorphometry

Vertebra (L_5_) was analyzed as described previously^26^. Briefly, for measurements of dynamic parameters, the mice were injected with calcein (i.p.) on day 1 and 8 before termination. The vertebrae were fixed in 4% paraformaldehyde, dehydrated in 70% EtOH and embedded in methyl meth-acrylate. The vertebrae were sectioned longitudinally and 4 μm-sections were stained with Masson-Goldner Trichrome for analyzing static parameters and unstained 8 μm-thick sections were analyzed for dynamic parameters. All parameters were measured using the OsteoMeasure histomorphometry system (OsteoMetrics) following the guidelines of the American Society for Bone and Mineral Research^27^.

#### Measurement of Mechanical Strength

Humerus was rinsed from muscle and stored in −20 °C until analysis. The three-point bending test (span length 5.5 mm) was performed at mid-humerus and the loading speed was 0.155 mm/s using a mechanical testing machine (Instron 3366, Instron). Based on the computer recorded load deformation raw data curves, produced by Bluehill 2 software v2.6 (Instron), the results were calculated with custom-made Excel macros.

### Cell Preparation and Flow Cytometry

Bone marrow cells were harvested from femur using a syringe with 5 ml of PBS. Pelleted cells were resuspended in Tris-buffered 0.83% NH_4_Cl solution to lyse erythrocytes, washed in PBS and resuspended in FACS buffer (PBS supplemented with 10% FCS (Sigma) and 0.1% NaN_3_). The total number of leukocytes was counted using Nucleocassettes and Nucleocounter (Chemometec). Cells were stained with PE-conjugated anti-CD19 (BD) and analyzed using a FACSVerse (Becton Dickinson). FlowJo software version 7.6.5 (Tree Star, Ashland, USA) was used for data analysis.

### Statistical Analyses

Values are given as mean ± sem. The statistical difference between placebo and E2 was calculated using Student’s *t*-test. The statistical differences in E2-response between WT and NOER mice were calculated by the interaction *P* value from a two-way-ANOVA analysis.

## Results

### Loss of membrane ERα signaling leads to disturbed hormonal feedback regulation

**Figure 1:**
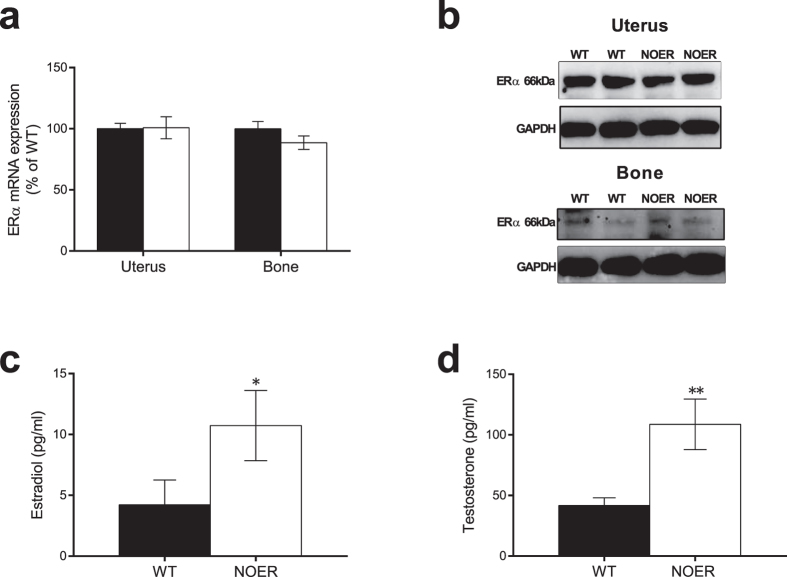
Loss of membrane ERα signaling leads to disturbed hormonal feedback regulation. ERα mRNA expression in uterus and bone from 16-week-old gonad intact female NOER and wild type (WT) mice (**a**). Western blot showing ERα protein expression in uterus and bone from NOER and WT mice (**b**) (blot images are cropped and full-length blots are presented in suppl. Fig. 1). Serum levels of 17β-estradiol (**c**) and testosterone (**d**), measured by GC-MS/MS in 12-week-old gonad intact female mice. Values are given as mean ± sem. [n = 10–14]. *p < 0.05, **p < 0.01, student’s *t*-test, NOER vs WT.

Gene expression level and protein expression of ERα was determined in uterus and bone and no differences were detected between NOER and control littermates (Fig. 1a,b). The ERα mRNA expression was slightly lower in the axial trabecular bone compared to the appendicular cortical bone, but this was observed both in WT (−29 ± 3%, p < 0.01) and in NOER mice (−25 ± 4%, p < 0.001). 12-week-old NOER mice displayed no differences in bone mass parameters, body weight, total body fat mass, or weights of liver, uterus or thymus compared to WT littermates (suppl. Table 1). To eliminate the possibility of a compensatory modulation of ERβ in NOER mice, ERβ gene expression was examined and found to be unchanged between WT and NOER mice in bone (suppl. Table 1). Importantly, serum levels of both 17β-estradiol and testosterone were significantly increased in NOER females compared to littermate controls (Fig. 1c,d), demonstrating a disturbed sex hormone feedback regulation in female mice lacking membrane ERα signaling. The fact that the skeleton was unaffected despite elevated sex steroid levels indicates that the phenotype data may be confounded and, therefore, in all subsequent experiments the estrogenic responses were evaluated in ovx mice. Ovariectomy resulted in an expected decrease in total body aBMD (WT: −9 ± 1%, p < 0.001; NOER: −5 ± 1%, p < 0.01), lumbar spine aBMD (WT: −14 ± 2%, p < 0.001; NOER: −9 ± 3%, p < 0.05), trabecular BV/TV (WT: −33 ± 3%, p < 0.001; NOER: −27 ± 4%, p < 0.01), and cortical thickness (WT: −9 ± 1%, p < 0.001; NOER: −7 ± 2%, p < 0.01) while no change by ovx was found on body weight.

### The estrogen response in the trabecular bone of the axial skeleton is strongly dependent on membrane ERα signaling

**Figure 2:**
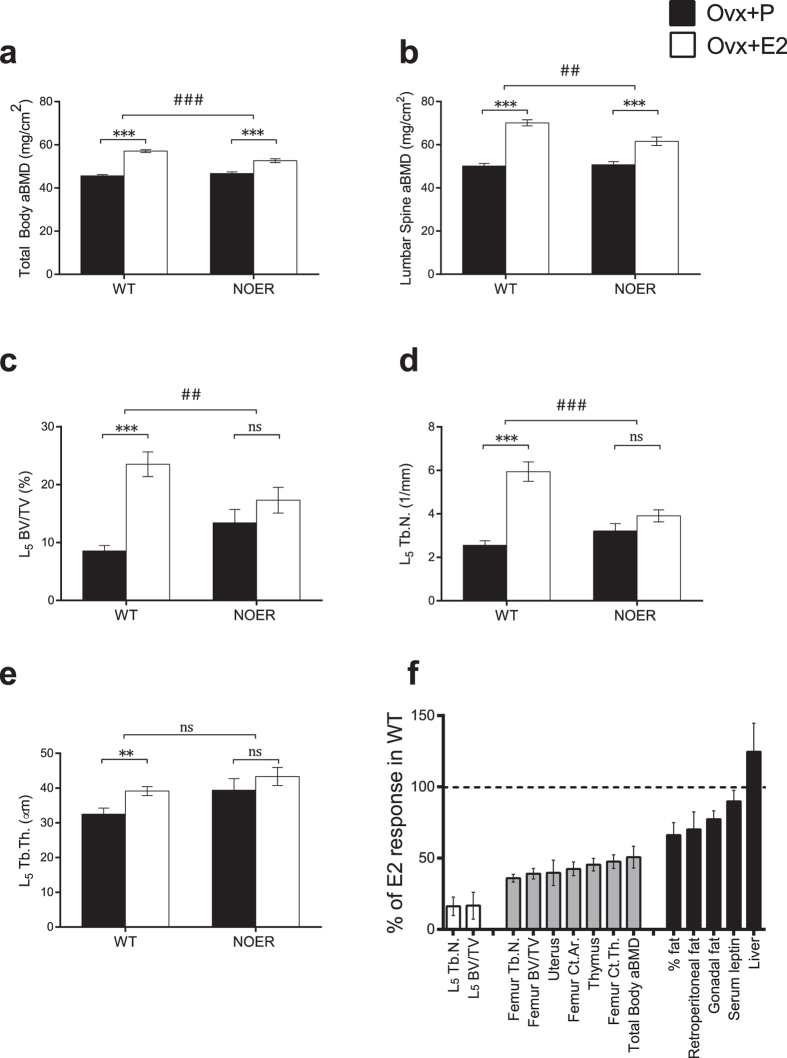
The estrogen response on trabecular bone mass in the axial skeleton is strongly dependent on membrane ERα signaling. 12-week-old NOER and wild type (WT) mice were ovariectomized and treated with 17β-estradiol (E2, 16,7 ng·mouse^−1^·day^−1^) or placebo (P) for four weeks. Total body (**a**) and lumbar spine (**b**) areal bone mineral density (aBMD) was measured by DXA. Trabecular bone volume per total volume (BV/TV) (**c**), trabecular number (Tb.N.) (**d**) and trabecular thickness (Tb.Th.) (**e**) were analyzed in vertebrae L_5_. The role of membrane ERα signaling for different tissues/parameters (**f**). The estrogenic response in WT mice, for each parameter, is set to 100%. The bars represent the estrogenic response in percent for the E2 treated ovx NOER mice compared to the estrogenic response in ovx WT mice, where 0% means no E2 response whereas 100% means normal E2 response. White bars; parameters with high (>80% reduction in E2 response) dependency on membrane ERα signaling, with no significant E2 effect in NOER mice. Grey bars; parameters with medium (40–70% reduction in E2 response) dependency on membrane ERα signaling, with significant E2 effects in NOER mice, but the E2 response is significantly attenuated when compared to the response in WT mice. Black bars; parameters with low or no dependency (<35% reduction in E2 response) on membrane initiated ERα signaling, with significant E2 effects in NOER mice that do not statistically differ from E2 effects in WT mice. Tb. N; trabecular number, BV/TV; bone volume per total volume, Ct; cortical, Th; thickness, Ar; Area, aBMD; areal bone mineral density. Values are given as mean ± sem. [n = 10–12]. **p < 0.01, ***p < 0.001, student’s *t*-test, E2 vs placebo treatment. ^##^p < 0.01, ^###^p < 0.001, interaction *P* value from two-way-ANOVA analysis, E2 effect in NOER vs E2 effect in WT.

Ovariectomized WT and NOER mice were treated with E2 or placebo for four weeks and the magnitude of the estrogen response was compared between WT and NOER females. Analysis of the skeleton by DXA showed an increase in total body areal bone mineral density (aBMD) after E2 treatment in both WT and NOER mice, compared to vehicle treatment (Fig. 2a). Interestingly, the estrogen response was significantly decreased in NOER females, compared to the estrogen response in WT littermates (−49%, p < 0.001, Fig. 2a,f). A similar pattern was seen after selective analysis of the lumbar spine aBMD where the estrogen response in NOER mice was attenuated when compared to the response in WT mice (−47%, p < 0.001, Fig. 2b). The estrogen response in the trabecular bone compartment of the axial skeleton was analyzed in more detail and E2 treatment, as expected, increased trabecular bone volume/tissue volume (BV/TV) in vertebrae in WT female mice (Fig. 2c). Importantly, the estrogen response was strongly attenuated in NOER mice (−83%, p < 0.01) compared to the response in WT mice and E2 treatment actually did not significantly increase trabecular BV/TV in the NOER mice (Fig. 2c,f), demonstrating a crucial role of membrane initiated ERα signaling for estrogenic effects on trabecular bone mass in the axial skeleton. The effect on BV/TV was mainly driven by an effect on vertebral trabecular number (Fig. 2d,e), which also displayed a pattern of strong dependency of membrane ERα (−84%, p < 0.001, Fig. 2d,f). Cortical thickness was also evaluated in the axial skeleton and this parameter was found to be significantly increased by E2 treatment in both WT (+50 ± 8%, p < 0.001) and NOER (+30 ± 5%, p < 0.001) mice. The E2 response on this cortical bone parameter was not significantly decreased in NOER mice compared with the E2 response in WT mice (−39%, non-significant). This is in contrast to the significant attenuation of the E2 response on vertebrae trabecular BV/TV (−83%, Fig. 2c) in NOER mice.

### The estrogen response in the appendicular skeleton is partly dependent on membrane ERα signaling

**Figure 3:**
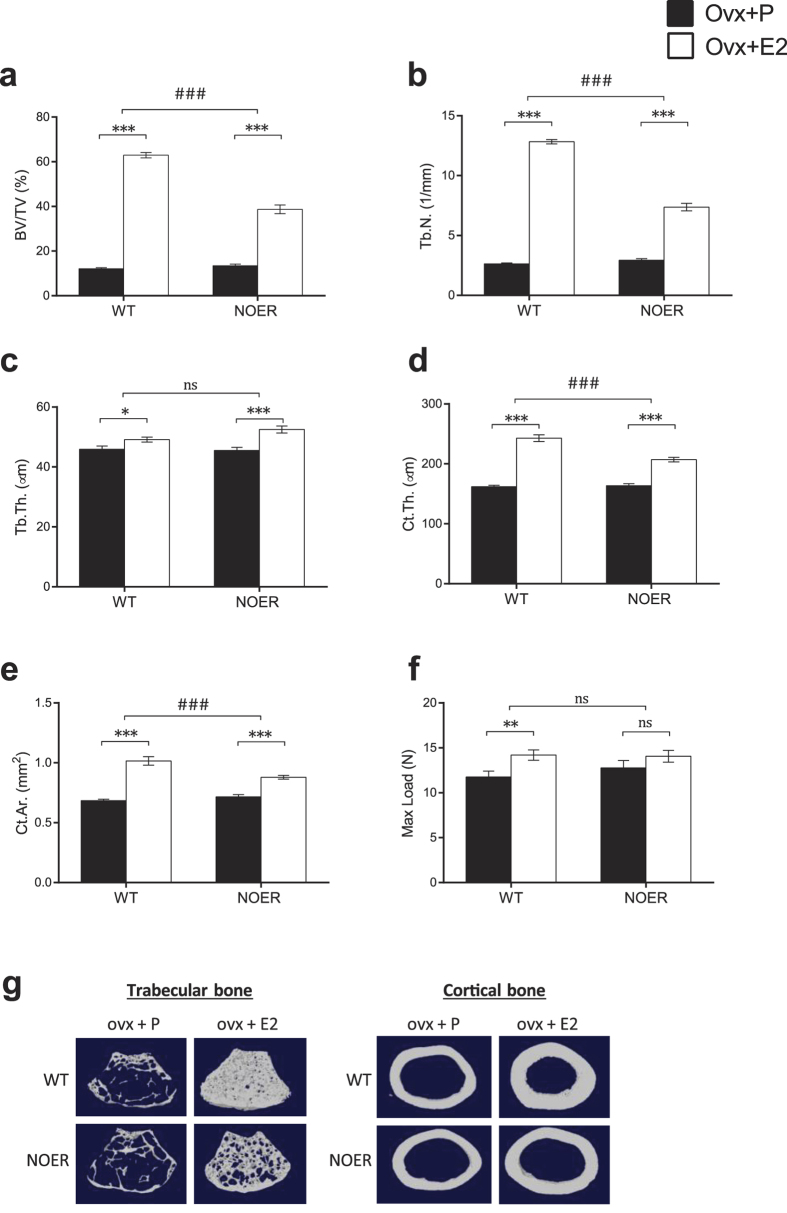
The estrogen response in the appendicular skeleton is partly dependent on membrane ERα signaling. 12-week-old NOER and wild type (WT) mice were ovariectomized and treated with 17β-estradiol (E2, 16,7 ng·mouse^−1^·day^−1^) or placebo (P) for four weeks. Trabecular bone volume per total volume (BV/TV) (**a**), trabecular number (Tb.N.) (**b**) and trabecular thickness (Tb.Th.) (**c**) were analyzed in the metaphyseal part of the distal femur. Cortical thickness (Ct.Th.) (**d**) and cortical area (Ct.Ar.) (**e**) were analyzed in the mid-diaphyseal part of the femur. Maximal load at failure (F_max_) (**f**) was analyzed by 3-point bending of humerus. Representative images of trabecular (left) and cortical (right) bone in femur (**g**). Values are given as mean ± sem. [n = 10–12]. *p < 0.05, **p < 0.01, ***p < 0.001, student’s *t*-test, E2 vs placebo treatment. ^###^p < 0.001, interaction *P* value from two-way-ANOVA analysis, E2 effect in NOER vs E2 effect in WT.

A thorough analysis of the long bones was performed to determine specific effects on the cortical and trabecular bone compartments. Analysis of the distal metaphyseal area of femur revealed a significant E2 effect on trabecular BV/TV in both WT and NOER mice but the estrogen response was significantly attenuated in NOER compared to the response in WT females (−58%, p < 0.001, Figs 2f and 3a,g). The reduced estrogen response on trabecular bone mass in NOER mice was mainly due to decreased estrogen response on trabecular number (−62%, p < 0.001, Figs 2f and 3b), while the effect of E2 treatment on trabecular thickness was similar between WT and NOER females (Fig. 3c). Analysis of the cortical bone compartment demonstrated increased cortical thickness as well as cortical area after E2 treatment in both WT and NOER littermates (Fig. 3d,e,g) and the estrogen responses were significantly attenuated in NOER mice compared to the responses in WT littermates both for cortical thickness (−53%, p < 0.001, Figs 2f and 3d) and area (−56%, p < 0.001, Figs 2f and 3e). The mechanical strength of long bones was analyzed by three-point bending and it demonstrated a significant increase in maximal load at failure after E2 treatment in WT females. However, no significant estrogen effect was seen in NOER mice as compared to vehicle treatment (Fig. 3f).

Histomorphometric analysis after four weeks of E2 treatment revealed no difference in E2 response between WT and NOER mice regarding osteoblast parameters (suppl. Table 2). However, the estrogen response on the osteoclast surface per bone surface differed between NOER and WT mice (suppl. Table 2), resulting in significantly higher osteoclast surface per bone surface in E2 treated NOER mice compared with E2 treated WT mice (+55%, p < 0.05, student’s *t*-test). No significant effects on dynamic bone parameters were found, most likely due to the establishment of a new steady state (suppl. Table 2).

### The importance of membrane ERα signaling is tissue-dependent

**Figure 4:**
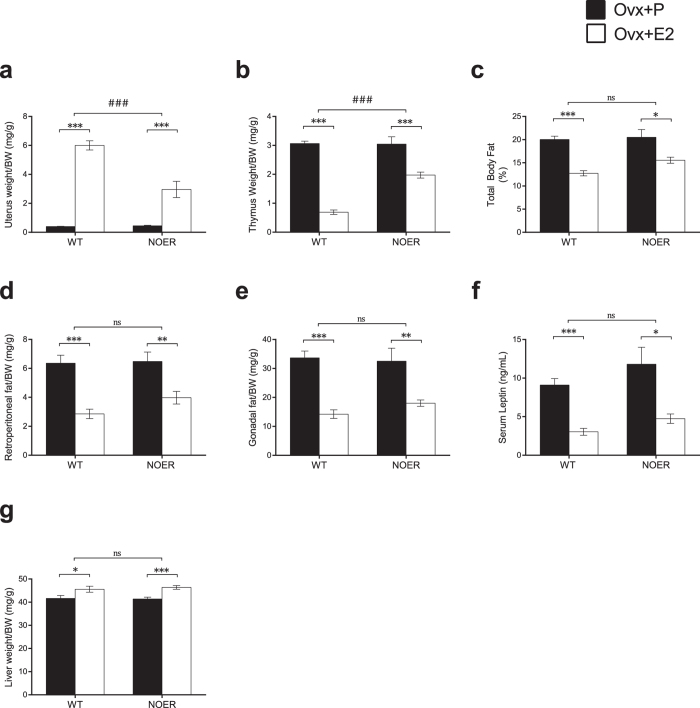
The importance of membrane ERα signaling is tissue-dependent. 12-week-old NOER and wild type (WT) mice were ovariectomized (ovx) and treated with 17β-estradiol (E2, 16,7 ng·mouse^−1^·day^−1^) or placebo (P) for four weeks. Estrogen effects on organ weights are given as weight per body weight (BW). Uterus weight (**a**), thymus weight (**b**), % total body fat measured by DXA (**c**), retroperitoneal fat weight (**d**), gonadal fat weight (**e**), serum leptin levels (**f**) and liver weight (**g**). Values are given as mean ± sem. [n = 10–12]. *p < 0.05, **p < 0.01, ***p < 0.001, student’s *t*-test, E2 vs placebo treatment. ^###^p < 0.001, interaction *P* value from two-way-ANOVA analysis, E2 effect in NOER vs E2 effect in WT.

To determine the tissue-dependent role of membrane ERα signaling, the estrogen responses in multiple well-known estrogen-sensitive tissues were investigated. Body weights were unchanged by E2 treatment after ovariectomy in both WT and NOER mice (suppl. Table 2). A significant estrogen effect on uterine weight was observed in both WT and NOER mice, but the estrogen response was partly attenuated in NOER mice (−60%, p < 0.001, Figs 2f and 4a). Analysis of the thymus revealed a significant reduction in thymus weight after E2 treatment, both in WT and NOER mice, and the estrogen response in NOER mice was partly decreased as compared to the response in WT mice (−55%, p < 0.001, Figs 2f and 4b). Estrogen treatment also significantly reduced the number of bone marrow cells and the frequency of B cells (CD19^+^ cells) in bone marrow in both WT and NOER mice (suppl. Table 2), and these estrogenic responses were also partly attenuated in NOER mice as compared to the responses in WT mice (−48%, p < 0.05 and −56%, p < 0.001 respectively, suppl. Table 2). Estrogenic effects on fat mass were determined by DXA measurements (% total body fat), dissection of fat depos (gonadal and retroperitoneal fat) as well as indirectly by serum leptin levels. All these parameters were significantly decreased by E2 treatment in both WT and NOER females (Fig. 4c–f), and there were no significant differences in estrogen responses between the two genotypes (Figs 2f and 4c–f). A similar pattern was seen for the E2 effect on liver weight, where the estrogen response did not differ between WT and NOER mice (Figs 2f and 4g), suggesting low or no dependency of membrane ERα signaling for the estrogenic effects on these parameters.

## Discussion

Estrogen signaling is important in several different tissues in the female body and increased knowledge regarding the tissue specific mechanisms behind these effects may aid in the search for tissue-specific estrogen treatments. To determine the role of membrane initiated ERα signaling in different estrogen responsive tissues, we have used genetically modified mice (NOER), in which ERα is incapable of localizing to the membrane. We, herein, demonstrate that membrane ERα signaling is of crucial importance for the estrogen response in trabecular bone in the axial skeleton, while the estrogen response in the appendicular skeleton is only partly dependent, and other parameters, including liver weight and total body fat mass, are independent of membrane ERα signaling. Thus, using a genetic approach we demonstrate a clear tissue-dependency for the role of membrane ERα in adult female mice.

We have studied mice in which the ERα cannot be palmitoylated at site C451 due to a point mutation, rendering the receptor unable to localize to the membrane^20^. In previous extensive studies, we have demonstrated that the NOER mice have no ERα in the plasma membrane fraction and their estrogen-stimulated rapid signal transduction is markedly deficient, consistent with absence of membrane ERα^20^,^28^. In addition, we here demonstrate that ERα levels were unaffected in bone and uterus of NOER mice, and we earlier showed that these mice have unaffected ERα levels in liver and mammary gland. Thus, although the NOER mouse model has no membrane localized ERα it has normal ERα levels in several estrogen responsive tissues, demonstrating that it is a valid model for the evaluation of the relative importance of membrane localized ERα for tissue-dependent estrogen responses.

It is well established that ERα is involved in the feed-back regulation of sex hormones in female mice^4^,^29^ and we here demonstrate that serum levels of both 17β-estradiol and testosterone, as analyzed by sensitive and specific GC-MS-technique^25^, were significantly increased in NOER mice, demonstrating that membrane ERα signaling is involved in the feed-back regulation of sex hormone levels in female mice. This finding is supported by previous findings of infertility and abnormal ovaries associated with elevated LH^23^ or increased 17β-estradiol^20^, in two separate mouse models devoid of membrane ERα^20^,^23^. Collectively, these studies clearly demonstrate that membrane localized ERα exerts effects *in vivo* and that it is necessary to evaluate the estrogenic response in ovx NOER mice, as the results in gonadal intact mice are confounded by elevated serum levels of sex hormones. Ovarian hormone levels, including testosterone and progesterone, decrease after ovariectomy alongside estradiol^25^ and are not replaced and we cannot rule out the possibility that the lack of interaction between estradiol and these other ovarian factors may influence our results, although we believe that this potential influence is minor.

**Figure 5:**
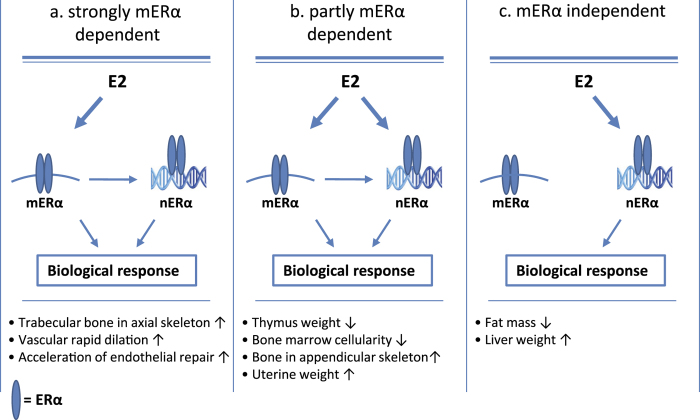
Proposed tissue-dependent role of membrane ERα (mERα) in multiple estrogen responsive tissues based on the findings in the present study and in a previous study regarding vascular estrogenic effects^23^, including (**a**) estrogen responsive tissues strongly dependent on membrane ERα, (**b**) tissues which are partly dependent on membrane ERα, suggesting cross-talk between membrane and nuclear ERα signaling and (**c**) tissues independent on membrane ERα signaling. **↑ = **estrogen increases**, ↓** estrogen decreases, nERα = nuclear ERα.

The knowledge of the complexity regarding ERα signaling has grown in recent years and thereby also the possibility to find tissue specific signaling mechanisms that may be targeted in the development of new selective estrogen modulators (SERMs). We recently showed that activation function 1 (AF-1) in ERα is important for estrogen response in bone and other tissues in a tissue-dependent manner^24^. We demonstrated that a functional AF-1 domain is important for the estrogen response in trabecular bone, while the response in cortical bone is AF-1 independent. Using the NOER mice, we here determined the role of specific cellular localization of ERα. The present study is the first to evaluate and compare the *in vivo* role of membrane ERα signaling in multiple estrogen responsive tissues. The main finding is that the estrogen responses in ovx NOER mice display a pronounced tissue-dependent pattern, including estrogen responsive tissues (i) strongly dependent on membrane ERα (trabecular bone in the axial skeleton), (ii) tissues which are partly dependent on membrane ERα (thymus weight, uterine weight and bone in the appendicular skeleton) and (iii) tissues essentially independent on membrane ERα signaling (total body fat mass and liver weight, Fig. 5). In addition, using a similar mouse model lacking membrane ERα, it is previously demonstrated that the estrogenic effects on certain estrogen-responsive vascular parameters (vascular rapid dilation and acceleration of endothelial repair) are dependent on membrane ERα signaling^23^, (Fig. 5a). Based on these findings, we propose that substances differentiating between membrane and nuclear ERα signaling might be useful as leads in the development of new selective estrogen modulators (SERMs) with improved tissue specificity profiles.

Membrane ERα signaling impacts transcription in several ways and several cell-culture studies propose important kinase-mediated cross-talk between membrane and nuclear ERα that modifies genomic responses to estrogen^23^,^30^,^31^. The DNA binding capacity is not affected by the C451A mutation but membrane ERα signaling is shown to be important for nuclear ERα transcriptional activity^20^. We propose that the ERα signaling in the tissues partly dependent on membrane ERα signaling may display a disturbed cross-talk between membrane ERα and nuclear ERα signaling (Fig. 5b).

Estrogen has profound effects on bone mass and is important both for growth of the skeleton and for regulation of bone remodeling in the adult skeleton^32^,^33^,^34^. We, and others, have previously demonstrated a crucial role of ERα for the estrogenic effects on bone mass in females^4^,^24^,^35^, while ERβ seems to have a more modulatory role^9^,^10^. The main tissue evaluated in the present study is the skeleton and the major finding is that membrane initiated ERα-signaling is crucial for the estrogen response on trabecular bone mass in the axial skeleton. Interestingly, the cortical bone in the axial skeleton was more modestly affected by loss of membrane ERα signaling compared to the trabecular bone, suggesting that trabecular and cortical bone are differently regulated by membrane ERα signaling in the axial skeleton. In addition, the estrogen response on the trabecular and cortical bone in the appendicular skeleton were partly dependent on membrane ERα, resulting in reduced estrogen response on mechanical strength of the long bones. Collectively, we have demonstrated that membrane ERα is critical for the estrogenic effects on bone mass with the most pronounced role in the trabecular bone in the axial skeleton. A role of non-nuclear ER signaling for estrogen effects on bone mass is supported by a study using an estrogen dendrimer conjugate (EDC), a macromolecule representing a modulated estrogen ligand that cannot enter the nucleus and thus only exerts non-nuclear estrogenic effects^17^. However, in that study EDC mainly increased cortical and not trabecular bone mass in ovx mice while mice devoid of membrane ERα had substantially reduced estrogen response in both the trabecular and cortical bone compartments. This difference might be explained by that EDC is a modulated estrogen ligand able to bind not only ERα but also ERβ in the membrane as well as the cytosol, while NOER mice only have disrupted membrane-localized ERα signaling.

It has been shown in osteoblasts, *in vitro*, that the estrogen response for approximately one third of all estrogen regulated genes is dependent on membrane ERα signaling^36^, indicating that, at least a part, of the membrane ERα initiated effect is primarily mediated via effects on osteoblasts. However, the present finding of increased osteoclast surface but unchanged bone formation in estrogen-treated NOER mice compared with estrogen treated control mice, indicates that the attenuated estrogen response in bone of NOER mice mainly is osteoclast-mediated. Nevertheless, it is possible that these effects on osteoclasts are indirect, initiated by ERα-mediated effects in osteoblasts.

Estrogen decreases fat mass via ERα^5^,^37^ and the estrogenic effects on adipose tissue include regulation of food intake and energy balance but also direct regulation of lipid synthesis in adipocytes^38^. We here show that the estrogen-induced decrease in total body fat mass is essentially independent of membrane ERα signaling. This finding is supported by the fact that fat mass in mice lacking nuclear localization of ERα is increased to a similar extent as in total ERα inactivated mice^39^, demonstrating a crucial role of nuclear ERα action for the regulation of fat mass. However, non-nuclear membrane initiated estrogen action has been shown to suppress lipid synthesis *in vitro* in mature adipocytes^28^. Thus, our *in vivo* data, showing that the estrogen effect on total body fat mass is essentially independent of membrane localized ERα, suggests that regulation of food intake and/or energy balance factors, but not lipid synthesis in adipocytes, seem to be the main mechanisms for nuclear ERα to regulate total body fat mass.

The liver is an estrogen-responsive organ and we found that the expected increase in liver weight after treatment with estrogen for four weeks was completely independent of membrane ERα signaling. Interestingly, Pedram *et al*. recently showed that membrane ERα signaling was required for normal regulation of genes regulating lipid and steroid synthesis in the liver^40^, suggesting that both membrane and nuclear ERα signaling is involved in the regulation of the liver.

The estrogen response on uterine weight in ovx mice lacking membrane ERα has previously been evaluated using two separate mouse models, revealing apparently opposite results^20^,^23^. In the present study, the estrogenic response on uterine weight in ovx mice was partly dependent on membrane ERα. We believe that these apparent differences in the role of membrane ERα for the estrogen response on uterine weight might at least partly be explained by the timing of ovx in relation to sexual maturation (before or after sexual maturation) and the magnitude of the response to the given estrogen treatment in WT mice. These findings also illustrate that to determine the tissue-specificity of estrogenic effects, it is critical to evaluate all estrogen-dependent phenotypes simultaneously, using identical conditions, as was done in the present study.

In conclusion, membrane initiated ERα signaling is important for the estrogen response in adult female mice *in vivo* in a tissue-dependent manner, and we show that membrane ERα signaling is crucial for the estrogen response in trabecular bone in the axial skeleton. Increased knowledge regarding membrane initiated actions of ERα may provide means to develop new selective estrogen modulators (SERMs) with improved profiles.

## Additional Information

**How to cite this article**: Gustafsson, K. L. *et al*. The role of membrane ERα signaling in bone and other major estrogen responsive tissues. *Sci. Rep.* **6**, 29473; doi: 10.1038/srep29473 (2016).

## Supplementary Material

Supplementary Information
