# Supplementary material for: The role of membrane ERα signaling in bone and other major estrogen responsive tissues
Source: Sci Rep. 2016 Jul 8;6:29473. doi: 10.1038/srep29473 (PMC4937452; doi:10.1038/srep29473)
Supplement: Supplementary Information [file srep29473-s1.doc]

**The role of membrane ERα signaling in bone and other major estrogen responsive tissues**

Gustafsson KL^1^, Farman H^1^, Henning P^1^, Lionikaite V^1^, Movérare-Skrtic S^1^, Wu J^1^, Ryberg H^1^, Koskela A^2^, Gustafsson J-Å^3^, Tuukkanen J^2^, Levin ER^4^, Ohlsson C^1*^ and Lagerquist MK^1*^

^*^Authors contributed equally

^1^Centre for Bone and Arthritis Research, Department of Internal Medicine and Clinical Nutrition at Institute of Medicine, Sahlgrenska Academy, University of Gothenburg, SE-41345 Gothenburg, Sweden

^2^Unit of Cancer Research and Translational Medicine, MRC Oulu and Department of Anatomy and Cell Biology, University of Oulu, FI-90014 Oulu, Finland

^3^Center for Nuclear Receptors and Cell Signaling, Department of Biology and Biochemistry, University of Houston, Houston, Texas, 77204-5056, USA

^4^Division of Endocrinology, Veterans Affairs Medical Center, Long Beach, California, CA92868, USA; Department of Developmental and Cell Biology, Department of Medicine, and Department of Biochemistry, University of California, Irvine, California, CA92868, USA

**Supplementary Table 1**

|  | **WT** | **NOER** |
| --- | --- | --- |
| **Body weight** **(bw)** (g) | 21,0 ± 0,3 | 21,6 ± 0,3 |
| **Uterus weight/bw** (mg/g) | 3,6 ± 0,5 | 2,6 ± 0,2 |
| **Liver weight/bw** (mg/g) | 41,9 ± 0,7 | 41,1 ± 0,7 |
| **Thymus weight/bw** (mg/g) | 2,4 ± 0,1 | 2,6 ± 0,1 |
| **Total body fat** (%) | 18,2 ± 1,0 | 20,0 ± 1,0 |
|  |  |  |
| **Total body BMD** (mg/cm^2^) | 50,0 ± 0,3 | 50,0 ± 0,5 |
| **Lumbar spine BMD** (mg/cm^2^) | 56,4 ± 1,3 | 54,1 ± 1,1 |
| **Trabecular BMD** (mg/cm^3^) | 236,6 ± 9,6 | 246,9 ± 13,4 |
| **Cortical thickness** (µm) | 182 ± 3 | 187 ± 3 |
| **Cortical area** (mm^2^) | 0,77 ± 0,01 | 0,79 ± 0,01 |
|  |  |  |
| **ERβ expression in bone** (AU) | 2,9 ± 0,2 | 2,7 ± 0,4 |

Body characteristics in 12-week-old and ERβ expression (trabecular bone) in 16-week-old gonadal intact female wild type (WT) and NOER littermates. Values are given as mean±sem. [n=10-14]. Student´s *t*-test, WT vs NOER mice. AU=arbitrary units.

**Supplementary Table 2**

|  | **WT** | | **NOER** | |
| --- | --- | --- | --- | --- |
|  | **Placebo** | **E2** | **Placebo** | **E2** |
| ***Body weight (g)*** | 22,2 ± 0,4 | 22,3 ± 0,5 | 23,2 ± 1,1 | 22,4 ± 0,6 |
|  |  |  |  |  |
| ***Serum markers*** | |  |  |  |
| **OCN** (ng/ml) | 110,6 ± 7,0 | 74,7 ± 5,4*** | 109,5 ± 9,5 | 64,0 ± 7,5** |
| **CTX** (ng/ml) | 27,0 ± 2,7 | 27,5 ± 1,5 | 24,8 ± 1,8 | 20,5 ± 2,6 |
|  | |  |  |  |
| ***Static histomorphometry*** | |  |  |  |
| **Oc.S/BS** (%) | 2,10 ± 0,20 | 2,03 ± 0,25 | 1,95 ± 0,23 | 3,15 ± 0,37*^#^ |
| **Ob.S/BS** (%) | 12,5 ± 1,0 | 6,3 ± 0,4*** | 10,9 ± 1,4 | 7,1 ± 0,7* |
|  | |  |  |  |
| ***Dynamic histomorphometry*** | |  |  |  |
| **MS/BS** (%) | 37,4 ± 2,1 | 34,3 ± 1,1 | 33,9 ± 1,9 | 32,4 ± 1,7 |
| **MAR** (µm/d) | 3,11 ± 0,39 | 2,58 ± 0,20 | 3,05 ± 0,35 | 2,56 ± 0,24 |
| **BFR/BS** (mm^3^/mm^2^/y) | 440 ± 66 | 324 ± 30 | 392 ± 58 | 301 ± 34 |
|  | |  |  |  |
| ***Bone marrow*** | |  |  |  |
| **Cellularity** (10^6^ cells/femur) | 6,2 ± 0,3 | 0,6 ± 0,2*** | 6,7 ± 1,0 | 3,6 ± 0,7*^#^ |
| **CD19^+^ cells** (frequency, %) | 36,5 ± 1,6 | 15,5 ± 2,2*** | 38,6 ± 2,4 | 28,8 ± 2,3*^#^ |

12-week-old NOER and WT mice were ovariectomized and treated with 17β-estradiol (E2, 16,7 ng·mouse^−1^·day^−1^) or placebo for four weeks. Values are given as mean±sem. [n=8-13]. *p<0.05, **p<0.01, ***p<0.001, student´s *t*-test, E2 vs placebo treatment. ^#^p< 0.05, interaction P value from two-way-ANOVA analysis, E2 effect in NOER vs E2 effect in WT. OCN; osteocalcin, CTX; serum cross-linked C-telopeptide of type I collagen, Oc.S/BS; osteoclast surface per bone surface, Ob; osteoblast, MS/BS; mineralizing surface per bone surface, MAR; mineral apposition rate, BFR/BS; bone formation rate per bone surface.

**Supplemental figure legend**

**Supplemental figure 1.** Western blot analyses of ERα protein in uterus and bone. Anti-GAPDH antibody was used as loading control. Protein from ERαKO uterus was added as negative control. WT uterus protein (Ut.) was added as positive control in c and d. a) ERα protein in uterus. b) GAPDH protein in uterus. c) ERα protein in bone. d) GAPDH protein in bone. L; Ladder, OL; overloaded uterus protein.
